# ﻿Integrative taxonomy of coastal *Cafiusbistriatus* (Erichson) species complex (Coleoptera, Staphylinidae)

**DOI:** 10.3897/zookeys.1100.79435

**Published:** 2022-05-12

**Authors:** In-Seong Yoo, J. H. Frank, Jong-Kuen Jung, Kee-Jeong Ahn

**Affiliations:** 1 Division of Restoration Research, Research Center for Endangered Species, National Institute of Ecology, Yeongyang 36531, Republic of Korea Division of Restoration Research, Research Center for Endangered Species, National Institute of Ecology Yeongyang Republic of Korea; 2 Entomology and Nematology Department, University of Florida, Gainesville, FL 32611, USA University of Florida Gainesville United States of America; 3 Department of Biology, Chungnam National University, Daejeon 34134, Republic of Korea Chungnam National University Daejeon Republic of Korea

**Keywords:** *Cafius*, coastal, *COI*, cryptic, *28S*, rove beetles, Staphylinidae, taxonomy

## Abstract

A systematic review of the marine littoral *Cafiusbistriatus* (Erichson) along the eastern Pacific and the western Atlantic coasts including the Caribbean Sea is presented based on morphological and molecular (*COI* and *28S*) characters. Specimens of the *Cafiusbistriatus* species complex [*C.bistriatus*, *C.rufifrons* Bierig, and *C.sulcicollis* (LeConte)] are similar to each other, including the form and structure of the aedeagus, and they can be treated as cryptic species. Detailed micromorphological characters (SEM) and molecular analyses support the validity of these three species. Intraspecific genetic divergence of *COI* using uncorrected p-distance among individuals of *Cafiusbistriatus* ranged from 0% to 1.56%, while interspecific divergence among three species ranged from 4.90% to 14.59%. All three species were each supported as a single lineage using *COI* and *28S* on both parsimony and maximum likelihood trees. Morphological characters among *C.bistriatus*, *C.rufifrons*, and *C.sulcicollis* are compared. *Cafiusbistriatusfulgens* Frank is synonymized under *C.bistriatus* and *Cafiusbistriatus* is redescribed with illustrations of diagnostic characters.

## ﻿Introduction

The marine littoral species *Cafiusbistriatus* (Erichson) occurs along the coasts of eastern Pacific and the western Atlantic including the Caribbean Sea and is known to be predaceous on invertebrates in decaying seaweeds on sandy beaches (Frank and Ahn 2011).

**Figure 1.**
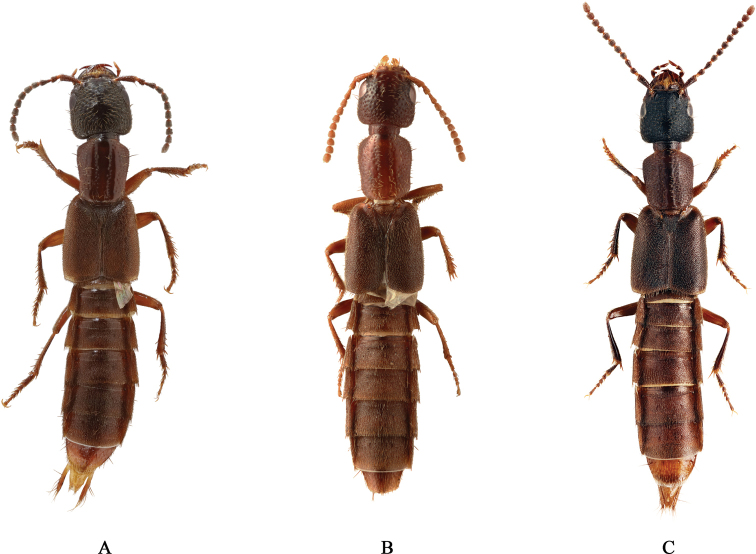
Habitus **A***Cafiusbistriatus* 6.7 mm **B***C.rufifrons* 6.3 mm **C***C.sulcicollis* 6.0 mm.

During a taxonomic revision of the genus *Cafius*, we recognized that *C.bistriatus*, *C.rufifrons* Bierig, and *C.sulcicollis* (LeConte) may be the same species. They are very similar in external form and internal structure including the male genitalia. Hereafter, we will refer to them as the *C.bistriatus* species complex (Fig. 1). These morphological similarities led us to study species delimitation of these three *Cafius* species more in detail.

We studied about 250 specimens of *Cafiusbistriatus* species complex (*C.bistriatus*, *C.rufifrons*, and *C.sulcicollis*) collected from a broad geographic distribution (Fig. 1). We also used additional molecular criteria based on genetic divergence and gene tree monophyly for species delimitation based on two loci (*COI* and *28S*) in order to test the accuracy of the species identifications.

In this paper, we compared morphological and molecular characters among individuals of the *C.bistriatus* species complex and redescribed *C.bistriatus* with illustrations of diagnostic characters.

## ﻿Materials and methods

For the study of morphological characters, we selected many specimens with different geographic distributions for their intraspecific variation, and dissected and cleared the whole body for examining microstructures (Yoo et al. 2021). Scanning electron microscope (**SEM**) and habitus photographs followed Yoo et al. (2021). Depository of specimens examined is as follows: The Natural History Museum (**BMNH**), London, United Kingdom; Canadian National Collection of Insects (**CNC**), Ottawa, Canada; Chungnam National University Insect Collection (**CNUIC**), Daejeon, Korea; Field Museum of Natural History (**FMNH**), Chicago, USA; Museum für Naturkunde (**MFNB**), Berlin, Germany; and Naturhistorisches Museum (**NMW**), Vienna, Austria.

For the study of molecular characters, we included a total of 18 specimens (18 for *COI* and 17 for *28S*) for DNA extraction in the dataset and the specimens studied are listed in Table 1. DNA extraction, sequencing, and alignments followed Song and Ahn (2018). The mitochondrial *COI* and nuclear *28S* were selected. Primers and amplification strategies are detailed in Yoo et al. (2021). Parsimony (**PA**) and maximum likelihood (**ML**) analyses, and intra- and inter-specific distances were conducted using search strategies described by Lee et al. (2020). Trees are rooted by *Cafiushistrio* (Sharp).

**Table 1.** List of species with their locality data and GenBank accession numbers. Asterisks and dashes indicate new addition and missing regions, respectively.

Species	Collection locality	*COI*	*28S*
*Cafiusbistriatus* 1	USA: Florida, Levy Co., Cedar Key	*OK398364	*OK398345
*Cafiusbistriatus* 2	USA: Florida, Monroe Co., Bahia Honda Key	*OK398365	*OK398346
*Cafiusbistriatus* 3	USA: Florida, Levy Co., Cedar Key	*OK398366	*OK398347
*Cafiusbistriatus* 4	USA: Florida, Levy Co., Cedar Key	*OK398367	*OK398348
*Cafiusbistriatus* 5	USA: New Hampshire, Rockingham Co.	*OK398368	*OK398349
*Cafiusbistriatus* 6	USA: Florida, Monroe Co., Florida Keys	*OK398369	*OK398350
*Cafiusbistriatus* 7	USA: Florida, Monroe Co., Florida Keys	*OK398370	*OK398351
*Cafiusbistriatus* 8	USA: Florida, Monroe Co., Bahia Honda Key	*OK398371	*OK398352
*Cafiusbistriatus* 9	USA: Florida, Monroe Co., Bahia Honda Key	*OK398372	*OK398353
*Cafiussulcicollis* 1	USA: California, Santa Barbara	*OK398373	*OK398354
*Cafiussulcicollis* 2	USA: California, Santa Barbara	*OK398374	–
*Cafiussulcicollis* 3	USA: California, Santa Barbara	*OK398375	*OK398355
*Cafiussulcicollis* 4	USA: California, Rufugio State Beach	*OK398376	*OK398356
*Cafiussulcicollis* 5	USA: California, Rufugio State Beach	*OK398377	*OK398357
*Cafiussulcicollis* 6	USA: California, Gaviota State Beach	*OK398379	*OK398358
*Cafiussulcicollis* 7	USA: California, San Simeon	*OK398378	*OK398359
*Cafiusrufifrons* 1	USA: Florida, Monroe Co., Keywest	*OK398380	*OK398360
*Cafiusrufifrons* 2	USA: Florida, Monroe Co., Keywest	*OK398381	*OK398361
*Cafiushistrio* 1	KOREA: Chungnam, Taean	MW407895	MW406746

## ﻿Results

### ﻿Molecular character analysis

In total, 35 new sequences from USA (California, Delaware, Florida, New Hampshire, North Carolina, and Puerto Rico) were generated (836 bp of partial *COI* gene region and *c.* 1034 bp of partial *28S*). All new sequences were deposited in GenBank (accession numbers: OK398364–OK398381 for *COI* and OK398345–OK398361 for *28S* in Table 1).

Intraspecific genetic divergence of *COI* using uncorrected p-distance among individuals of *Cafiusbistriatus* ranged from 0% to 1.56%, while interspecific divergence among three species ranged from 4.90% to 14.59% (Table 2). All three species were each supported as a single lineage using *COI* and *28S* on both PA and ML trees (Figs 6, 7).

**Table 2.** Inter- and intraspecific genetic differences in *Cafiusbistriatus*, *C.rufifrons*, and *C.sulcicollis* for *COI* (836 bp) calculated using p-distance.

	*C.bistriatus*	*C.rufifrons*	*C.sulcicollis*
*C.bistriatus*	0–1.56		
*C.rufifrons*	11.72–14.59	0	
*C.sulcicollis*	4.90–6.10	13.76–14.59	0.12–1.20

### ﻿Taxonomy

#### ﻿Genus Cafius

Taxon classificationAnimaliaColeopteraStaphylinidae

Stephens

A6492975-C82B-5DF0-B5D9-3305EB4FBDEA

Cafius Stephens, 1829: 23 (Type species: Staphylinusxantholoma Gravenhorst).

#### ﻿ Cafius bistriatus

Taxon classificationAnimaliaColeopteraStaphylinidae

(Erichson)

384B21D1-2C9E-5431-8610-AD080A03030E

**Figure 2.**
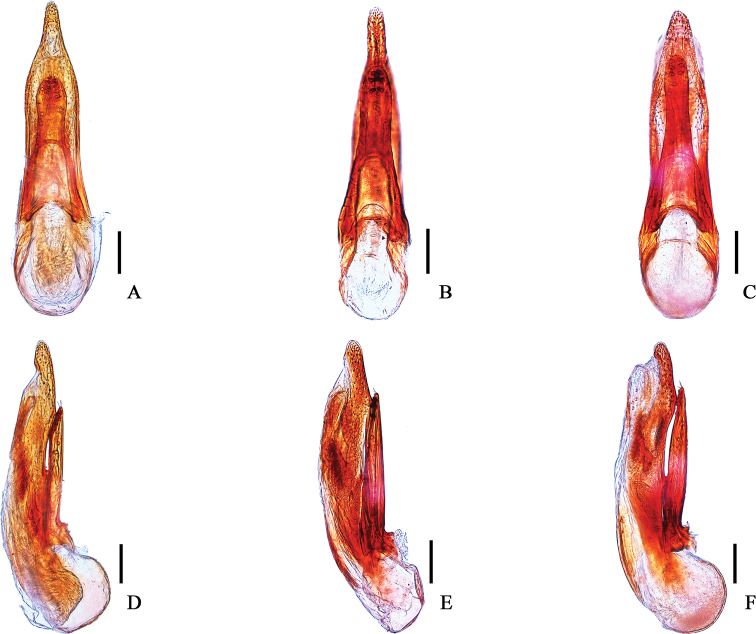
Aedeagi **A, D***Cafiusbistriatus***B, E***C.rufifrons***C, F***C.sulcicollis***A–C** lateral aspect **D–F** dorsal aspect. Scale bar: 0.1 mm.

**Figure 3.**
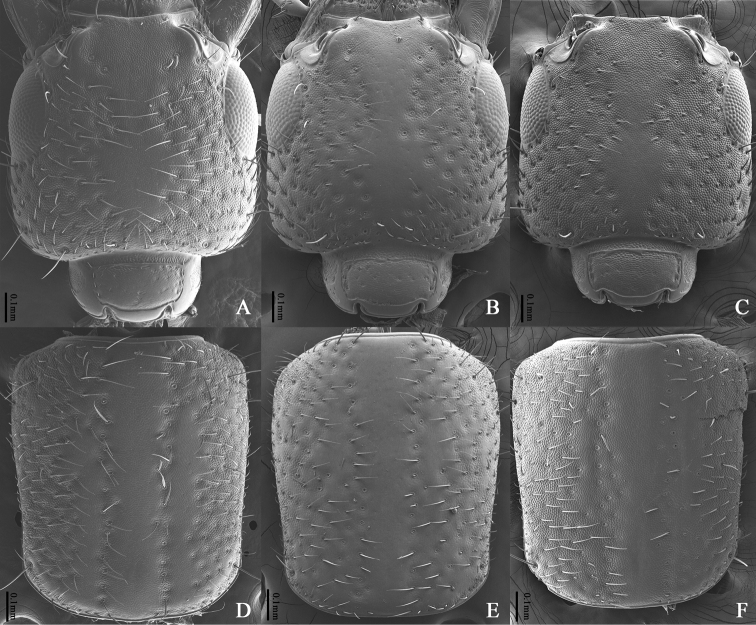
SEM photographs **A, D***Cafiusbistriatus***B, E***C.rufifrons***C, F***C.sulcicollis***A–C** head, dorsal aspect **D–F** pronotum, dorsal aspect.

**Figure 4.**
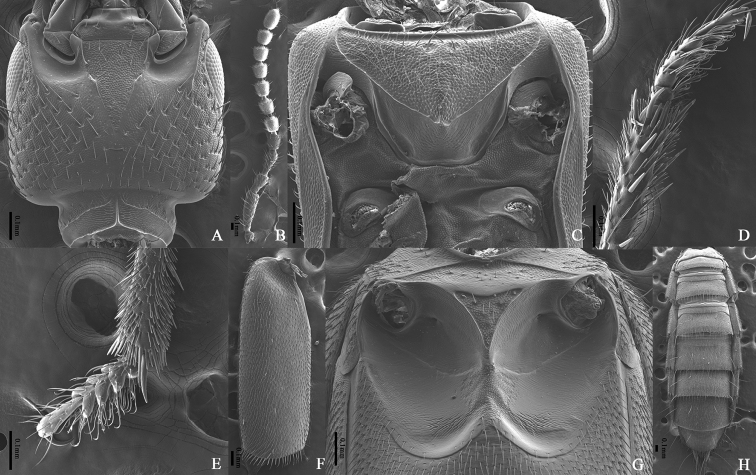
*Cafiusbistriatus***A** head, ventral aspect **B** antenna **C** prosternum, ventral aspect **D** front tibia, lateral aspect **E** front tarsus, dorsal aspect **F** elytron, dorsal aspect **G** meso- and metaventrites, ventral aspect **H** abdomen, dorsal aspect.

**Figure 5.**
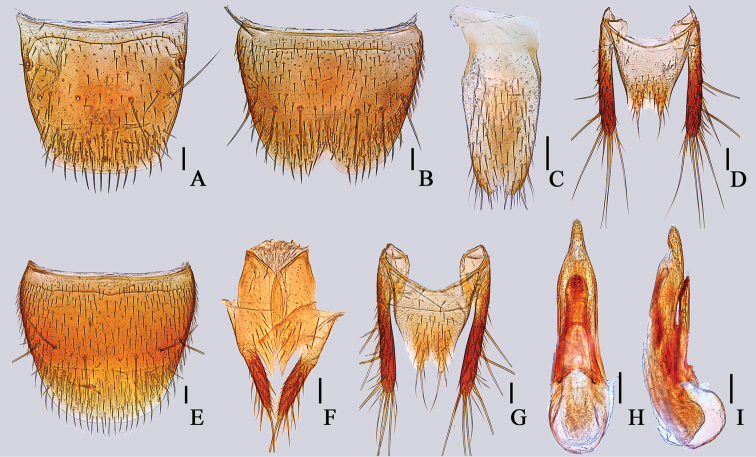
*Cafiusbistriatus***A** male tergite VIII, dorsal aspect **B** male sternite VIII, ventral aspect **C** male sternite IX, ventral aspect **D** male lateroteral sclerite and tergite X, dorsal aspect **E** female sternite VIII, ventral aspect **F** gonocoxite, ventral aspect **G** female tergite X, dorsal aspect **H** aedeagus, dorsal aspect **I** aedeagus, lateral aspect. Scale bar: 0.1 mm.

Figs 1A , 2A, D , 3A, D , 4 , 5

Philonthus bistriatus Erichson, 1840: 502. [Type locality: Americae septentrionalis Insula Longa (Long-Island); 1 syntype in MFNB].
Philonthus bilineatus Erichson, 1840: 503. [Type locality: Americae meridionalis Insula Antiguae, St. Johannis (St. John’s, the capital of Antigua); 1 syntype in MFNB].
Cafius bistriatus fulgens Frank, 1986: 153 [in Frank et al. 1986] (Cafius; subspecies of bistriatus). [Type locality: Mexico: Baja California Sur: Mulege]. new synonym. See Herman (2001) for the detailed synonymy.

##### Type specimens examined.

1 syntype, “6155 || *bilineatus* | Typus | Er. f. 503. || Antigua | Moritz 5.5 || *Philonthus* | *bilineatus* || = *bistriatus* Er.” (MFNB); 1 syntype, “6152 || *bistriatus* | Typus | Er. f. 502 || Long. 75(2. | Zimmerman | 5.5. || = *bilineatus* Er. | sec. Fauvel || *bistriatus* | Er.” (MFNB).

##### Other specimens examined.

**Canada**: 6 exx., Nova Scotia, Cape Breton H. N. P. Presqu’île, 3 m, PG562728, 13.IX.1984\J. M. Campbell & A. Davies, sifting beach wrack; 1 ex., Nova Scotia, Cape Breton H. N. P. Ingonish N. Bay, PG984711\29.VI.1983, L. LeSage, LL54 TP18, seashore wrack; 2 exx., Nova Scotia, Cape Breton H. N. P. Pleasant Bay\27.V.1984, L. Masner, seabeach kelp; 2 exx., Nova Scotia, Cape Breton H. N. P. Pleasant Bay, seashore kelp\29.VII.1983, D.E., J.E. Bright pans; 4 exx., N. S. Cape Breton Highl. N. P. 25 km SE Cap Rouge, 14.VI.84, A. Smetana; 1 ex., C. I.\Schwarz; 1 ex., New Brunswick, Passamaquoddy Bay, Pottery Bch., 29.VII.1976, M.J. Dadswell; 1 ex., New Brunswick, Passamaquoddy Bay, Pottery Bch., 29.VII.1976, M.J. Dadswell; 1 ex., QUE: St. Adelaide 0.5 mi. W. Sandy Beach Stn., 21.VIII.1953, E.L.Bousfield; 3 exx., QUE., 4 mi. S. Riviėre-à-Claude, VII-18-1972, 200’, J.M. Campbell; 5 exx., N. S. Point Aconi, VIII-13-1972, J.M. & BA Campbell; 5 exx., N. S. Big Bras d’Or, VII-25-1972, J.M. Campbell; 1 ex., Kouchibouguac N. P., N. B., 1.VI.1977, S.J. Miller, Code-5195U\*Cafiusbistriatus* Er. Det. J.M. Campbell 1978; 1 ex., Kouchibouguac N. P., N. B., 13.IX.1977, J.M. Campbell, Code-5953Y. **Jamaica**: 2 exx., JA., Clarendon P., Jackson’s Bay, 12.XII.1972, J. Peck\Sifting algae on beach; 8 exx., JA., St. Ann Runaway Bay, 22.VIII.1974, S. Peck, beach drift; 10 exx., JA. St. Catherine, 7mi. SE. Sp. town, Hellshire Beach, 27.VII.1974, S. Peck, beach drift; 1 ex., JA., Trelawny Parish, Duncans, VIII.21.1966, Howden & Becker. **Mexico**: 6 exx., Mulege SEAWEED Baja Cal. Sur, Mex., J. Klink Coll., X.3.66\ERIC; Scheerpeltz; 48 exx., Sonora, NR. PT. Cirio, 29.50-112.40, 28 VIII 1974 V. Roth Coll; 163 exx., Sonora, EI Desemboque, 29.30-112.34, 23 V 1974, Brown & Speich ex flying on beach; 2♂2♀, Sonora, EI Desembogue May 23 1974 Brown & Speich; 1 ex., Veracruz Prov. 8km S Veracruz, Hwy 150, 10 July 1990 J. Ashe, K-J. Ahn, R. Leschen ex: under seaweed on beach; 1 ex., Bahia Magdelena, 1 June 1968 W. G. Evans ex; under turtle carapace\SM0038043 KUNHM-ENT. **USA**: 1 ex., 698., A30 S. Thomas, *bistriatus* Er. S. Thomas, coll. L.W. Schaufuß; 1 ex., Marion, Mass. Bowditch., *Cafiusbistriatus* Er., Coll. Schubert; 7♂6♀ (2♂1♀ dissected, 5♂5♀ in 100% EtOH, AC220), Puerto Rico, Municipio Fajardo, Seven Seas Beach, 18°22.227'N, 65°38.359'W, 6 VI 2009, Park09-021, JS Park, *ex* under seaweeds; 4♂10♀ (1♂ dissected, 2♂9♀ in 100% EtOH, AC250), Florida, Pinellas Co., Anclote Gulf Park, 25 X 2008, KJ Ahn, JH Ahn, under seagrasses; 1♂5♀ (1♂ dissected, 5♀ in 100% EtOH), Florida, Brevard Co., Port Canaveral Jetty Park, 14 II 2009, KJ Ahn, under stones with empty barnacles; 2 exx., Florida Tampa, Clearwater Beach, 10 VI 1995, K.-J. Ahn., ex under seaweed; 14 exx. (2♂ dissected, 8 in 100% EtOH), Florida, Cedar Key, 28 XII 2015, KJ Ahn; 1 ex., FLA., Flamingo, Everglades Nat. Pk., 4.I.1971, L. Masner; 1 ex., CONN, Fairfield Co. Norwalk, 9 Aug 78 Calf Pasture Beach\R.E.Orth family\Wrack-debris on sandy beach; 2 exx., CONN, Fairfield Co. Westport, 11 Aug 78 Sherwood Is. St. Pk\R.E.Orth family\Wrack-debris on sandy beach\M3; 1 ex., CONN, Fairfield Co. Fairfield, 10 Aug 78 Jennings Beach\R.E.Orth family\Wrack-debris on sandy beach; 34 exx. (1♂ dissected, 33 in 100% EtOH), North Carolina, Dare Co., Oregon Inlet, 6 IV 2009, KJ Ahn, JH Ahn, under seaweeds; 2 exx., Cape Cod, Massachusetts, VII.3.1975, E. J. Kiteley, under clumps seaweed Beach; 1 ex., South Shore S. I., N. Y.\C. L. Pollard, Apr. 11-111, No.; 1 ex., Portland Maine, July 23 1966, E. J. Kiteley, dry full carcass; 1 ex., C. I.\145\Schwarz; 1 ex., Md.; 1 ex., Pt. Isabel, Texas, VI-26-30\JOMartin Collector; 2 exx., Lynn, Mass. Essex Co. V -12; 2 exx., Lynn, Mass. Essex Co.; 1 ex., N.Y. Rockaway L.I., 15.V.1941 W. Spector\C.N.H.M. 1960 Boris Malkin Coleoptera Colln.; 2 exx., Barnegat Bay NJ JW Green VIII.4.28 \C.N.H.M. 1960 Boris Malkin Coleoptera Colln.; 1 ex., Rocwy Bcb. L. L.; 1 ex., Peekskill 4/8.80 NY\Sherman; 1♀\Marion Mass. Bowditch.\Cafiusbistriatus Er.\ex coll. Scheerpeltz\bistriatus Er.; 1 ex., TEXAS: Wilacy Co. Port Mansfield 30 September 1990 J. S. Ashe ex., beach wrack.

##### Redescription.

Body medium sized, length 5.5–7.7 mm, forebody length (from clypeus to end of elytron) 3.1–3.7 mm. Body brown to dark brown; head black; metaventrite dark brown to black; anterior part of epipleura usually brighter than elytra. **Male. *Head*** (Figs 3A, 4A). Dull, subquadrate with rounded hind angles, as long as wide (Length/Width = 1.00). Microsculpture reticulate on median, intermediate, and dorso-lateral region; partly coalescent on ventral region; granulate on submentum and gular region. Short longitudinal linelike depression absent on interocular region. Punctures on dorsal surface large and pit-like, median longitudinal impunctate region moderate in size and longitudinal. Seta absent on median region; more or less evenly distributed on intermediate, lateral, and ventral region. Frontoclypeal setiferous puncture, preocular setiferous puncture, lateral ocular setiferous puncture, four occipital setiferous punctures, genal setiferous puncture, and infraorbital setiferous puncture present; interocular setiferous punctures absent; postocular setiferous puncture closer to posterior margin of head than posterior margin of eye. Mentum with two pairs of long setae. Gular sutures completely converged. Dorsal basal carina and nuchal carina present on neck. Antenna (Fig. 4B) filiform, not short, exceeding posterior margin of head; antennomeres 1–3 very elongate, 4–8 elongate, 9–10 slightly elongate; approximate length ratio of each antennomere 60: 30: 29: 23: 25: 25: 26: 24: 23: 22: 26. Compound eye large, longer than half length of temple (Eye length/Temple length = 0.81–0.86); interfacetal setae very short. ***Mouthparts***. Labrum with each lobe moderately transverse, U-shaped; transparent apical membranous part less broad than sclerotized part. Mandibles asymmetrical; blade present, not making small tooth on blade; left and right one both with two internal teeth; 6–7 gland serial pores present. Maxillary palpomere 4 more or less fusiform, narrower than penultimate one. Labial palpomere 3 more or less fusiform; ligula slightly emarginate, seta absent. ***Thorax***. Pronotum (Fig. 3D) glossy on median region, dull on lateral region; rectangular, longer than wide (Length/Width = 1.18–1.19); lateral margin slightly sinuate. Microsculpture indistinctly reticulate on median region; transverse on intermediate region; granulate on lateral region. Disc with homogeneous punctures similar to those of head in size; seta absent on median and intermediate region; densely distributed on lateral region; median impunctate region not clearly defined and not elevated, but depressed series of dorsal setiferous punctures present on midline forming 2 longitudinal rows, each with about 20 punctures, not separated distinctly from ground punctures; largest setiferous lateral puncture separated from lateral carina by at least 3 times width of the puncture. Prosternum (Fig. 4C) with 2 long macrosetae on central region; sternacostal carina making angle posteriorly. Hypomeron with distinct microsculpture but seta absent. Elytra (Fig. 4F) long (Length/Width = 2.25–2.42), longer than pronotum at midline (Elytron length/Pronotum length = 1.42–1.50), wider than pronotum in maximum width (Elytra width/Pronotum width = 1.41–1.53); one subhumeral seta present but lateral seta absent; punctation simple and dense. Mesoventrite (Fig. 4G) with transverse carina connected to lateral margins of mesoventral process; mesoventral process more or less pointed; mesocoxal cavities narrowly separated, posterior margin completely developed. Front tibia (Fig. 4D) with several spines; front tarsomeres 1–4 (Fig. 4E) strongly dilated laterally, tarsomere 5 broadened apically, pale setae slightly spatulate; hind tarsomere 1 longer than 5. ***Abdomen***. Microsculpture reticulate on tergites (Fig. 4H); punctures more or less coalescent; pubescence on each tergite more or less longitudinally directed. Posterior margin of segments III–VI straight. Posterior transverse basal carina complete on tergites III–VI, absent on tergite VII. Pubescence of segment VIII much sparser than segment VII. Tergite VIII (Fig. 5A) with 2 long macrosetae present on each side of midline, apical margin arcuate. Laterotergal sclerite (Fig. 5D) long and slender, with 5 long macrosetae and 2 long macrosetae on tip. Posterior margin of tergite X emarginate apically, apical portion pigmented. Apical setae of tergite X present. Basal carina on sternite III rounded. Sternite VIII (Fig. 5B) with 3 long macrosetae on each side of midline, apical margin deeply emarginate. Basal part of sternite IX (Fig. 5C) well developed, asymmetrical; posterior margin of sternite IX emarginate. ***Aedeagus*** (Fig. 5H, I) Apical process of median lobe abruptly narrowed on ventral aspect, not constricted in apical third. Paramere longer than half length of median lobe; apical region rounded on ventral aspect; about 7 acorn-shaped pegs present on apico-medial region, forming more or less 2 rows; 4 apical and 2 pairs of lateral setae present on margin, apical setae separated from lateral setae, all setae similar in length. **Female.** Apical margin of abdominal sternite VIII (Fig. 5E) entire, rounded; gonocoxite II narrowly tubular (Fig. 5F); gonostyle with 1 long macroseta on tip, separated from gonocoxite II and sclerotized. Tergite X as in Fig. 5G.

##### Distribution.

Canada, USA (Pacific and Atlantic coasts), Mexico, West Indies, Venezuela.

##### Remarks.

Members of the *Cafiusbistriatus* species complex (*C.bistriatus*, *C.rufifrons*, and *C.sulcicollis*) are very similar in external form and internal structure and so they could be treated as cryptic species. A comparison table of morphological characters among *C.bistriatus*, *C.rufifrons*, and *C.sulcicollis* is presented in Table 3.

## ﻿Discussion

The diagnostic characters among members of the *Cafiusbistriatus* species complex (*C.bistriatus*, *C.rufifrons*, and *C.sulcicollis*) include sculpture and punctation patterns on the head and pronotum, and form and structure of the male genitalia. Blackwelder (1943) first noted that *C.rufifrons* was very similar to *C.bistriatus*. However, Orth and Moore (1980) noted that these two species are different in morphological characteristics such as the shape of the median lobe and head color. Also, they mentioned differences in sculpture and punctation patterns on the pronotum between *C.bistriatus* and *C.sulcicollis* on the Pacific coast. Frank et al. (1986) discussed the differences in head sculpture, body size, and the shape of male genitalia between *C.bistriatus* and *C.rufifrons* on the Atlantic coast. They also described Pacific coast populations of *Cafiusbistriatus* as the separate subspecies, *C.b.fulgens* Frank, mainly based on the microsculpture of head but it is more similar to that of *C.rufifrons*. No differences in male genitalia were mentioned. We synonymized this subspecies under *Cafiusbistriatus* because they are within the variation of *C.bistriatus* such as the form of the aedeagus and the sculpture patterns on head and pronotum.

We have studied all of these species (Fig. 1) as well as substantial series of specimens throughout the range of USA (California, Delaware, Florida, New Hampshire, North Carolina, and Puerto Rico), Canada, Mexico, West Indies and Venezuela. Specimens of *Cafiusbistriatus* from both Atlantic and Pacific coasts were examined, though no sequences were available from the Pacific coast. Members of the *Cafiusbistriatus* species complex (*C.bistriatus*, *C.rufifrons*, and *C.sulcicollis*) very closely resembled each other in external and internal form and structure including the aedeagus (Fig. 2). More importantly, however, the shape (including length and width) of the paramere and median lobe of the male aedeagus are different in each species, and they each should be considered as valid. For example, the paramere of *C.bistriatus* is less elongate compared to that of *C.sulcicollis* and the median lobe of *C.rufifrons* is narrower than those of both. See Table 3 for more differences among these three species.

**Table 3.** Comparison of morphological characters among *Cafiusbistriatus*, *C.rufifrons*, and *C.sulcicollis*. See Fig. 2 for sculpture patterns of head and pronotum.

	*C.bistriatus*	*C.rufifrons*	*C.sulcicollis*
Head color	black or almost black	reddish brown	black
Head sculpture on median region	reticulate, meshes with flat cells	slightly reticulate, broken meshes	heavily reticulate, meshes with convex cells
Median impunctate region on head	moderate in size	broader	narrower
Pronotum sculpture on median region	slightly reticulate	glossy	dull and reticulate
Median lobe on dorsal aspect	broader	narrower	broader
Paramere	shorter, stout narrower than median lobe	intermediate, stout, as wide as median lobe	longer, slender, much narrower than median lobe
Distribution	both Atlantic and Pacific coasts of North America	southern Florida-USA, Cuba	California-USA

**Figure 6.**
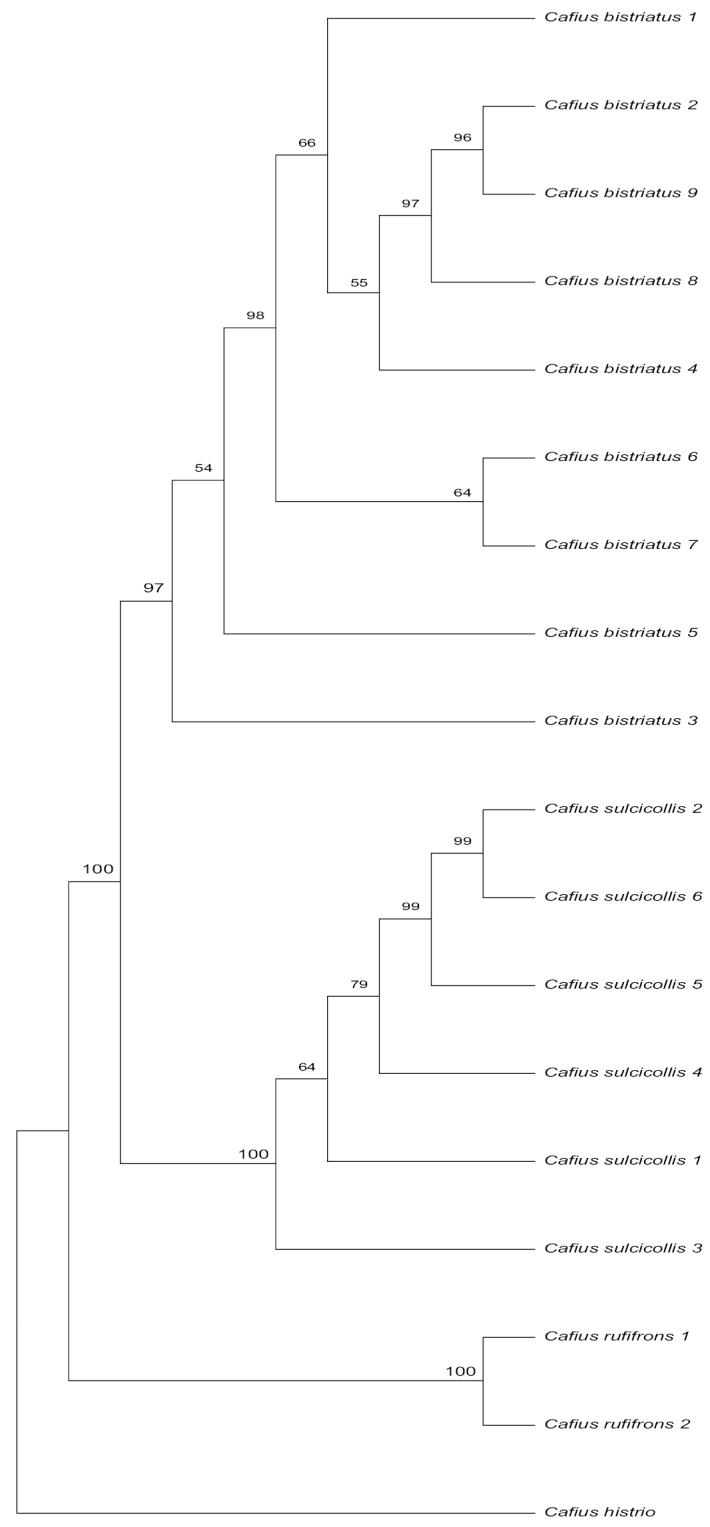
Parsimony tree of *Cafiusbistriatus* and its related species based on partial *COI* and partial *28S* gene sequences with bootstrap values.

**Figure 7.**
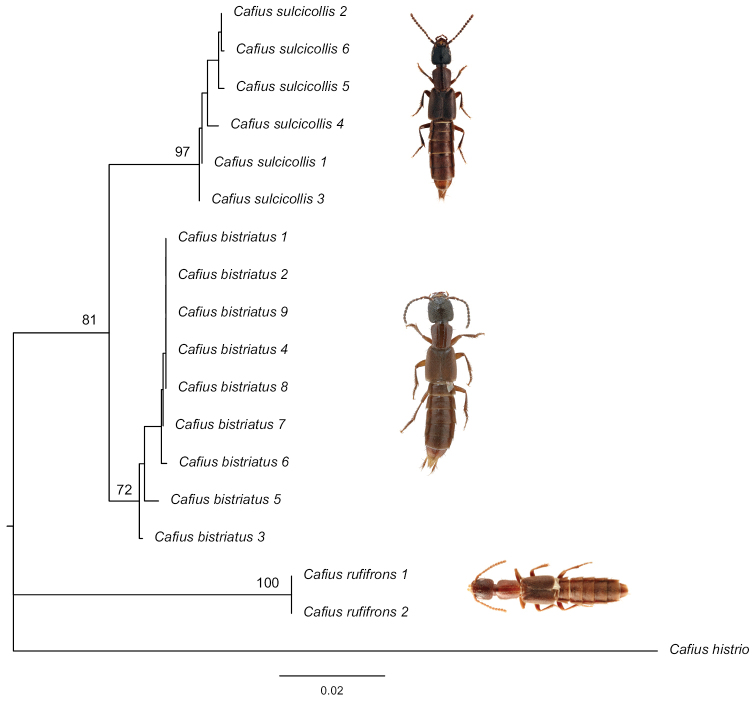
Maximum likelihood tree of *Cafiusbistriatus* and its related species based on partial *COI* and partial *28S* gene sequences with bootstrap values.

In addition, we used the criteria of genetic divergence and gene tree monophyly for correct identification and species delimitation of these three *Cafius* species. Intraspecific genetic divergence of *COI* using uncorrected p-distance among individuals of *Cafiusbistriatus* ranged from 0% to 1.56%, while interspecific divergence among three species ranged from 4.90% to 14.59% (Table 3). Recently, Lee et al. (2021a) showed that intraspecific divergence of *COI* ranged from 0.00% to 2.51% for six Korean *Cafius* species. Our results are within the range of this study although our dataset is relatively small compared to theirs. In addition, *Cafiusbistriatus*, *C.rufifrons*, and *C.sulcicollis* are each formed as distinct lineages on PA and ML trees (Figs 6, 7). Their phylogenetic relationships were not fully resolved based on *28S* data (not shown) but they are fully resolved based on *COI* data (not shown) and the concatenated data of *28S* and *COI* (Figs 6, 7). Therefore, we consider all of them as valid species and also because they are also different in morphological characters, as discussed above.

*Cafiusbistriatus* occurs on both Atlantic and Pacific coasts of North America and its dispersal depends on sea currents and winds as in *C.algarum* (Sharp) (Lee et al. 2021b). *Cafiusbistriatus* may be introduced to the western Atlantic from the eastern Pacific coasts, probably through the Panama seaway (unpublished data).

Unfortunately, only two individuals of *C.rufifrons* for *COI* and *28S* sequences were available in this study, and no individuals were available for the Pacific population of *C.bistriatus*. For a more robust test of species limits within the *C.bistriatus* complex, we suggest future work to include more intensive and broader sampling from the oceanic beaches along the eastern Pacific and western Atlantic coasts.

## Supplementary Material

XML Treatment for Cafius

XML Treatment for Cafius bistriatus

## References

[B1] Blackwelder RE (1943) Monograph of the West Indian beetles of the family Staphylinidae. Bulletin - United States National Museum 182(182): 1–658. [i–viii] 10.5479/si.03629236.182.i

[B2] Erichson WF (1840) Genera et species Staphylinorum insectorum coleopterorum familiae. Zweiter Band. F. H. Morin, Berlin, 401–954. 10.5962/bhl.title.59644

[B3] Frank JH Ahn KJ (2011) Coastal Staphylinidae (Coleoptera): A worldwide checklist, biogeography and natural history. ZooKeys 107: 1–98. 10.3897/zookeys.107.1651 PMC3392188 22792029

[B4] Frank JH Carlysle TC Rey JR (1986) Biogeography of the seashore Staphylinidae*Cafiusbistriatus* and *C.rufifrons* (Insecta: Coleoptera). Florida Scientist 49(3): 148–161.

[B5] Herman LH (2001) Catalog of the Staphylinidae (Insecta, Coleoptera): 1758 to the end of the second millennium. Bulletin of the American Museum of Natural History 265: 1–4218. 10.1206/0003-0090.265.1.1

[B6] Lee J Lee JS Ahn KJ (2020) Reappraisal of Korean *Oxyporus* Fabricius (Coleoptera: Staphylinidae: Oxyporinae), including a new species based on morphological and molecular characters. Journal of Asia-Pacific Entomology 23(3): 680–688. 10.1016/j.aspen.2020.04.012

[B7] Lee JS Shin YJ Ahn KJ (2021a) Molecular taxonomy of Korean *Cafius* Stephens, 1829 (Coleoptera: Staphylinidae: Staphylininae) with description of a new species. The Pan-Pacific Entomologist 97(2): 45–54. 10.3956/2021-97.2.45

[B8] Lee JS Yoo IS Ahn KJ (2021b) A taxonomic review of coastal *Cafiusalgarum* (Sharp) (Coleoptera: Staphylinidae) with two new synonyms and discussion of its distributional extension. Coleopterists Bulletin 75(4): 835–844. 10.1649/0010-065X-75.4.835

[B9] Orth RE Moore I (1980) A revision of the species of *Cafius* Curtis from the west coast of North America with notes of the east coast species (Coleoptera: Staphylinidae). Transactions of the San Diego Society of Natural History 19(13): 181–211.

[B10] Song JH Ahn KJ (2018) Species trees, temporal divergence and historical biogeography of coastal rove beetles (Coleoptera: Staphylinidae) reveal their early Miocene origin and show that most divergence events occurred in the early Pliocene along the Pacific coasts. Cladistics 34(3): 313–332. 10.1111/cla.12206 34649372

[B11] Stephens JF (1829) The nomenclature of British insects; being a compendious list of such species as are contained in the Systematic Catalogue of British Insects, and forming a guide to their classification, &c. &c. Baldwin and Cradock, London, UK, 68 pp. 10.5962/bhl.title.51800

[B12] Yoo IS Lee JS Ôhara M Ahn KJ (2021) Three synonyms of the coastal *Phucobius* Sharp species (Coleoptera: Staphylinidae) are proposed based on morphological and molecular characters. Journal of Asia-Pacific Entomology 24(1): 320–328. 10.1016/j.aspen.2020.12.015

